# Preferred Tone of Nutrition Text Messages for Young Adults: Focus Group Testing

**DOI:** 10.2196/mhealth.4764

**Published:** 2016-01-19

**Authors:** Christina Mary Pollard, Peter A Howat, Iain S Pratt, Carol J Boushey, Edward J Delp, Deborah Anne Kerr

**Affiliations:** ^1^ School of Public Health Curtin University Bentley Australia; ^2^ Department of Health, Western Australia Perth Australia; ^3^ Cancer Council Western Australia Perth Australia; ^4^ School of Psychology and Speech Pathology Curtin University Perth Australia; ^5^ Epidemiology Program University of Hawaii Cancer Center Honolulu, HI United States; ^6^ Department of Nutrition Science Purdue University West Lafayatte, IN United States; ^7^ School of Electrical and Computer Engineering Purdue University West Lafayette, IN United States

**Keywords:** text messages, tone of voice, nutrition messages, fruit, vegetable, junk food, alcohol, communication

## Abstract

**Background:**

Young adults are a particularly hard to reach group using conventional health promotion practices as they do not see nutrition messages as personally relevant to them. Text messaging (short message service, SMS) offers an innovative approach to reaching young adults to support and promote dietary behavior change.

**Objective:**

The aim of this study was to develop and test tonal preferences for nutrition text messages among young adults using focus groups.

**Methods:**

A total of 39 young adults aged 18-30 years residing in Perth, Western Australia participated in four focus groups. Participants briefly discussed their perception of healthy eating and their responses to messages about increasing fruit and vegetables, and reducing “junk food” and alcohol intake. They ranked their preference for 15 nutrition messages across 3 dietary behaviors (fruit and vegetables, junk food, and alcohol) with 5 different message tones (authoritative, empathetic, generation Y, solutions, and substitutions) and identified the messages most likely to persuade young adults to change their diet. A 5-point ranking of the nutrition messages was from the most likely to least likely to persuade (1-5). The focus groups were conducted by a trained facilitator and observer and were recorded. Data driven content analysis was used to explore themes. Tonal preferences and potential motivators were collated and frequencies presented.

**Results:**

Participants ranked offering substitutes (29%, 11/39) and using empathy (22%, 9/39) as the most persuasive message techniques in improving diets of young adults, with low responses for Generation Y (17%, 7/39), solutions (17%, 7/39), and authoritative (15%, 6/39) tones. Females were more likely to consider substitution messages persuasive (35%, 7/20) compared with males (22%, 4/19). A greater proportion of males compared with females considered authoritative messages persuasive: (22%, 4/19) compared with (7%, 1/20). There is a strong preference for a substitution tone for fruit and vegetable messages (52%, 20/39), and no overall message tone preference for junk food and alcohol messages. Substitutions were viewed as helpful and practical. Empathy was liked as it acknowledged previous efforts. Responses to authoritative tone were mixed with some feeling guilt while others found them informative. Acceptability of the solutions depended on the behavioral change and acceptability of the solution proposed. Generation Y tone had some support for junk food and alcohol messages, and if favored, was considered casual, humorous, catchy, and motivational.

**Conclusions:**

Substitutions and tone of empathy were favored as the most likely execution styles to motivate nutrition behavior change across all participants. There is no “one size fits all” with different tones preferred by individuals for different dietary behaviors. Although text messaging provides instant message delivery direct to the individual, these results demonstrate the complexity of developing motivational nutrition message for young adults. These findings reveal the importance of considering the tone and content and pretesting messages for health promotion text message interventions.

## Introduction

Dietary guidelines established from scientific evidence provide credible and reliable nutrition information for health professionals and policy makers to underpin nutrition interventions [1,2]. Public health nutrition priorities include encouraging populations to increase their fruit and vegetable intake, decrease their energy dense nutrient poor (EDNP) food and beverage intake, and reduce alcohol consumption [3,4]. The challenge is how to translate this complex information into messages that consumers find relevant. Health authorities have embraced social media tools, such as text messaging (short message service, SMS), to expand the reach of health communications, foster engagement, and increase access to credible, science-based health messages [5,6]. Message development, implementation, and evaluation should be viewed as central to any campaign designed to influence health behavior [7]. Message persuasiveness is related to the source of the message, recipient characteristics, and context as well as the particular desired outcome (eg, attitude, intention or behavior) [8].

Commercial companies, public sector bodies, and charities use a brand “Tone of Voice” (ToV) in their communications to engage people with messages regarding their products and services [9]. The ToV conveys the nature of the brand personality in a way that is accessible and liked by consumers, for example, a friendly or official tone. The strength of the message recipients’ impression of the brand can be related to message expressions describing feelings or actions that are used to engage the audience and are largely based on recipients taking action. Companies strive for distinct brand values developed through linguistics; for example warm, expert, and friendly. However, other than through sales and awareness, little is known about the impact of message ToV on the message recipient in terms of health behavior change. The impact of the message ToV for the specific target audience is important because it can vary and evoke differing responses [8].

Young adults are a particularly hard to reach group using conventional health promotion practices as they do not see nutrition messages as personally relevant to them and may be less likely to take notice of conventional health promotion approaches [10]. Text messaging may better engage young adults; however, the messages need to be short and to the point, with 140-160 characters the recommended message length [5]. The advantage of text messaging is that it is delivered directly to the individual and is likely to be read within minutes of receiving [6]. To date, health-related text messages have been used in physical activity, weight loss, and smoking interventions [11-16] but have not been fully evaluated in population-based approaches in nutrition [17]. Text messaging interventions to encourage better nutrition have the potential as a cost-effective rapid communication method with high population reach.

Achieving the correct tone, content, and length of messages for text message delivery is challenging and the complexity of message development needs to be considered [18]. A criticism of physical activity text messaging interventions is that the majority of studies failed to provide information on how the text messages were developed [15]. Prior to conducting a text messaging intervention, important steps are needed to carefully construct the messages and undertake pretesting to avoid potential unintended effects [14].

Young adults aged 18-30 years were the target for the Connecting Health and Technology (CHAT) randomized controlled trial (RCT) to improve nutrition behaviors using mobile devices and tailored text messaging in young adults, desribed in full elsewhere [19]. In brief, young adults were the target as they had particularly poor intakes of fruit and vegetables, a high intake of energy dense nutrient poor food, and an excessive alcohol intake due to binge drinking. There was some evidence of misconceptions relating to what constituted a healthy diet among young adults and at the time, little was known about the best approach to motivate dietary change in this group. The mobile phone application for dietary intake measurement addressed issues of respondent burden and increased engagement in this traditionally hard-to-reach population who are high users of mobile phones. The ultimate goal of the CHAT RCT was to deliver messages that were persuasive and motivated dietary behavior change. The objective of this study was to develop and test tonal preferences for short nutrition messages among young adults using focus groups as formative evaluation for the CHAT RCT.

## Methods

### Participants

Young adults aged 18-30 years were recruited through social media (Facebook), emails sent to workplaces, and snowballing techniques in October to December 2010. They were invited to take part in a 90 minute focus group discussion about food and eating at an advertising agency in an accessible central metropolitan location. Those responding to the advertisements were screened for eligibility and booked into a focus group. Exclusion criteria were focus group participation in the previous 6 months and studying or working in advertising, marketing or health-related industries. After conducting four focus groups, the facilitator and observer assessed that no new information was emerging. Focus groups were segmented by gender and age (18-22 years, 23-30 years) to facilitate discussion and identify any gender and age group differences as well as to minimize social desirability bias. All participants received written information about the study and signed individual consent forms. The Curtin University Human Ethics Committee approved the study.

### Message Development

Short motivational messages were developed by the investigators, who are accredited practicing dietitians and health promotion experts, to persuade behavioral changes in 3 areas (to *increase* fruit and vegetable consumption and to *decrease* alcohol and EDNP [or junk food] intake). Each message was 2-3 sentences long to make it suitable for delivery via a text message. Next, an advertising agency copywriter applied 5 different tonal executions to each of the 3 areas based on ToV approaches used in commercial advertising. The 5 tonal executions were authoritative, substitution, solution, empathetic, and generation Y (Gen Y). Table 1 provides an explanation of each ToV. The number of messages was limited to 15 as this was the maximum number that time would permit for in-depth discussion during the focus groups. The 15 final “test” messages applied the 5 ToV approaches to a message to increase fruit and vegetables, reduce junk food, and reduce alcohol intake. These final messages were ranked by participants on their potential to motivate behavioral change. Tone and content preferences were explored through focus group discussions.

**Table 1:** Definitions used for five different tonal executions of messages explored in the focus groups.

Five tone of voice	Definition
Authoritative	An all-knowing nutrition expert, telling them what and what not to do with educational reasoning
Empathetic	Sympathetic tone that says you understand the struggles and indulgences they have and are just trying to be informative and helpful
Gen Y engaging	Speaking in their language using peer-to-peer slang, as if you are their best buddy telling them cool news or insights on a personal one-to-one level to develop affinity with the subject
Solutions based	Providing tips and ideas to encourage them to eat more healthy food, by showing them how
Substitution based	Tips and ideas or showing how to choose a healthier option (swap, don’t stop)

### Focus Groups

The investigators prepared focus group outlines to test general influences on diet, preferred ToV and content for text messages. A female facilitator and male observer with extensive advertising experience moderated the focus groups. The investigators had no contact with participants other than during screening and observed the focus groups from another room via a video screen. Focus groups were held in the evening to enable those working to attend. All groups were audiotaped with participants informed that they were being observed and recorded. The facilitators explained the research aims and informed the participants that the investigators were planning to use text messaging to motivate people to change their diet. The discussion started with broad questions about what participants thought people their age ate, their motivations for food choices, and what would make them change their food choice. This first set of questions is referred to as general influencers of food choice and message preference in the results section.

To assess message tone, each participant was asked to rank their preference regarding the persuasiveness of 5 messages in each of the 3 nutrition behavioral change areas. For each behavioral change area, they were provided with the message in the 5 tonal executions resulting in a list of the 15 messages as shown in Table 2. The facilitator then handed out the list of messages in the 5 different ToV. Participants individually ranked the 5 tonal messages according to their potential to persuade young adults to change their diets from 1 (favorite) to 5 (least favored) for each behavior change area. An in-depth discussion about the messages took place following the ranking. The preference for words or phrases used in the messages and suggestions for alternatives were noted during focus groups.

**Table 2:** Focus group participants rating of behavior change persuasiveness of tonal messages, n=39.

Nutrition behavior change area	Tone of voice	Message and abbreviation	Rated as most likely to persuade change (%)
Fruit and vegetables	Authoritative	You need lots of fruit and vegetables to stay healthy. If you’re not getting 2 serves of fruit and 5 serves of veg every day, you are just not looking after yourself. (FVAut)	11
	Empathetic	Two fruit and five veg every day sounds like hard work, but when you get into it, you’ll find it’s easy. You’ll be less likely to snack on junk, and you’ll feel great! (FVE)	21
	Gen Y	Fruit and veg make awesome healthy snacks, and they stop you craving nasty fatty stuff. The guys in white coats reckon 2 serves of fruit and 5 serves of veg every day is the go. (FVGY)	5
	Solution	Do a daily fruit and veg shop, and you’ll never go without. You’ll always have something healthy to whip up in the kitchen, and you’ll eat less junk. (FVSol)	11
	Substitution	It’s not hard to work in more fruit and veg every day. Swap fruit for other snacks, roast veggies for chips, and have a fruit salad for breakfast. (FVSub)	52
Junk food	Authoritative	Junk food is high fat, salt, and sugar and low in nutrition. So avoid it. (JFAut)	10
	Empathetic	We’re all tempted by a “quick-fix” of junk food now and again. You really should be strong and say NO! (JFE)	23
	Gen Y	It’s called junk food coz it’s no good for you! It’s all bad. You know you feel better if you leave it alone.(JFGY)	18
	Solution	Make sure you’ve always got a few days’ fresh food in the house. You won’t get caught out and end up eating junk food if you’re prepared for a healthy diet.(JFSol)	28
	Substitution	There’s always a tasty alternative to junk food. Swap an apple for a piece of cake, veggies for chips, have salads for snacks, and a yummy sanger instead of that burger.(JFSub)	21
Alcohol	Authoritative	Not only is alcohol full of useless energy that can make you fat, it is harmful even in the smallest amounts. Listen to the health experts and avoid alcohol, or if you insist on drinking, limit your intake.(AAut)	23
	Empathetic	Most people like a drink now and again, but unfortunately alcohol is a killer when it comes to weight gain. Try and remember the empty calories when someone offers you a drink.(AE)	21
	Gen Y	You booze-you lose. Unfortunately not weight though. Alcohol is packed with totally pointless calories. It’s also pretty damn bad for you.(AGY)	28
	Solution	If you’re trying to maintain a healthy weight drop the drink. Alcohol is deceptively fattening. Drink less and try and acquire a taste for mineral water!(ASol)	13
	Substitution	Alcohol is full of pointless calories so slow down when you go out. Drink less and swap alcoholic drinks for mineral water or juice.(ASub)	15

### Data Analysis

The audio recordings were transcribed and combined with the observers’ detailed notes. Themes were identified in accordance with the method described by Owen [20]. Data-driven content analysis was used to explore the findings with 3 researchers identifying themes independently. The investigators and facilitators reviewed the responses and confirmed the main themes and specific phrases that demonstrated them. Quantitative data on tonal preference ranking were collated and frequencies reported. For the purpose of reporting, each topic area was given an abbreviation code relating to the message content: Junk Food (JF); Fruit and Vegetables (FV); and Alcohol (A); Authoritative (Aut); Substitution (S); Solution (Sol); Empathetic (E); and Generation Y (GY). The abbreviation for the message and the tone were combined to assist the reader in identifying individual messages by tone, eg, the authoritative fruit and vegetable message is (FVA). A list of abbreviations is given in Table 3.

**Table 3:** List of message tone abbreviations.

Abbreviation	Description
FVAut	Fruit and vegetable authoritative
FVE	Fruit and vegetable empathetic
FVGY	Fruit and vegetable Gen Y
FVSol	Fruit and vegetable solution
FVSub	Fruit and vegetable substitution
JFAut	Junk food authoritative
JFE	Junk food empathetic
JFGY	Junk food Gen Y
JFSol	Junk food solution
JFSub	Junk food substitution
AAut	Alcohol authoritative
AE	Alcohol empathetic
AGY	Alcohol Gen Y
ASol	Alcohol solution
ASub	Alcohol substitution

## Results

### Participants

Four focus groups were conducted with a total sample of 39 participants (19 males and 20 females). Each group comprised 9 or 10 participants. Participant characteristics are shown in Table 4. The majority were in paid employment and had either completed or were enrolled in tertiary studies. The results are presented in four sections: the first section was a general discussion on influencers on food choices and text messages to promote nutrition; the second section provides a short summary of the ranking of likelihood for message tone to motivate change, shown in Table 5; the third, presentation of the major themes regarding response to the tone and potential influence of each message; and the last section is the summary of preferences for words and phrases, Table 6; and final messages developed for the intervention, Table 6.

**Table 4:** Characteristics of young adults participating in focus group sessions.

Demographics		n (%)
**Gender**		
	Males	19 (%)
	Females	20 (%)
**Employment status**		
	Not working/unemployed	3 (7.7)
	Working	26 (66.7)
	Student	10 (25.6)
**Level of education**		
	Completed secondary school or less	9 (23.1)
	Completed university (or currently completing)	30 (76.9)
**Household income**		
	≤US $60,000 per annum	7 (17.9)
	≥ US $60,000 per annum	32 (82.1)
**Sample**		
	Sample total	n=39
	Mean age in years (SD)	23.2 (2.9)

### Section 1: General Influencers of Food Choice and Message Preference

#### Convenience, Price, and Feelings

When discussing general influences on young adults’ food choices, convenience, price, and feeling or emotions were mentioned as the main influencers. Convenience was important as participants described competing demands on their time. The struggle to fit in both work and study left limited time to prepare food. Healthier food was also considered more expensive than junk food.

...Unhealthy fast food is a lot cheaper than the healthy food I like, I could go for a salad, but it’s probably quicker and cheaper to buy something junky.

Feelings influenced food choice; it just depends on how they feel on that day and what they want to eat. There was a general view that message credibility depended on who delivered the communication, with government health departments and nongovernment health organizations (eg, Cancer Council or Heart Foundation) considered credible sources of nutrition information. Most participants were aware of health recommendations regarding alcohol, fruit and vegetables, and body weight. Specific action communications, (eg, “avoid weight gain, avoid junk food”) and those with additional explanation were favored. The issue of alcohol intake and weight gain was particularly salient for younger women but only a few men.

#### Unrealistic Alcohol Recommendations, but Want to Know More

Alcohol drinkers viewed current alcohol recommendations as unrealistic for their lifestyle “When you go out to drink you drink a lot more.” The motivators for drinking were considered very different than those described in the messages, “All of these miss the point, we don’t drink for nutrition.” and “it’s fun, makes you feel good or better.” Social norms and peer pressure were seen as the main barriers to cutting down on alcohol consumption, “drinking is normal–socially impressive.”

Identifying motivators to change drinking habits was challenging but “feeling ill the next day” appeared to resonate overall as did the use of memorable slogans, and health statistics highlighting the consequences of drinking too much. Many were interested in the long term harm associated with excessive alcohol intake.

#### Text Messages Mode Appreciated, but Length and Tone Important

Delivering messages via a text was appreciated, but the timing and tone was important, (eg, “common knowledge but good to have in this form”). Some messages were “too long” and “preachy” but agreed the content was true. Tone was also important, “sounds like a message from mum”. Message timing was considered crucial to curb junk food intake, (eg, “If it comes too late you will have already bought the junk food”).

### Tonal Preferences

Across the board participants ranked offering substitutes (29%, 11/39) and using empathy (22%, 9/39) as the most persuasive message techniques in improving diets of young adults, with low responses for Gen Y (17%, 7/39), solutions (17%, 7/39), and authoritative (15%, 6/39) tones. Females were more likely to consider substitution messages persuasive (35%, 7/20) compared with males (22%, 4/19). Also, a greater proportion of males (22%, 4/19) compared with females (7%, 1/20) considered authoritative messages persuasive.

Table 5 shows that there was no clear overall tonal preference across all messages with the exception of the substitution ToV which was ranked as most likely to motivate change in fruit and vegetables by 52% of participants. The preferred tone for junk food and alcohol messages were more diverse, 28% chose the solution tone for junk food and the same proportion chose Gen Y for alcohol messages. The main themes emerging from the focus group discussions on message feedback are reported by ToV with examples relating to the specific messages identified with the abbreviations shown in Table 3.

### Authoritative Tone

#### Condescending yet Provoking, a Good Reminder and Informative

There were mixed reactions to the authoritative messages. Some considered them condescending, boring or even offensive, provoking feelings of guilt or a defensive response. Yet most said they were informative and made them listen. The intensity of reaction to the authoritative tone depended on the nutrition message.

The authoritative FVAut message reinforced known health authority messages and was viewed as “Positive” and an effective reminder “Makes you want to eat 2 fruit and 5 veg.” For others, FVAut was considered strong but informative “A bit harsh. It is needed but it can also offend” or “True, makes you think I guess”. Some had the same opinion for authoritative alcohol AAut, “Good info but might offend people”.

#### Junk Food Tone Strong

For some, junk food, authoritative JFAut was stating the obvious but not necessarily effective, “We know this but it doesn't stop us.” For others it was “straight to the point”, “informative” or as having “shock value” reminding them about how bad junk food was. The “so avoid it” wording was effective and led some participants to ask for some clarification and more information about the health consequences, “Being told to eat less is a powerful message” and “The first sentence is effective but should finish with an outcome, eg, Prolonged use will cause…[what?]’’.

#### Alcohol Tone Offensive yet Informative

Some responded negatively to the AAut message, “Feels like someone is telling you off, I would ignore this one.”, and clarity was needed by some “Does it want us to stop or limit?” A common view was that although the message might make people realize that alcohol was not good for them, it would not stop them drinking but may help limit their intake, “If it said limit their intake then I would be more inclined to adhere to it.” There was poor knowledge and disbelief about the harmful health effects of drinking alcohol and some participants requested more medical information. One participant defended the benefits of “some” alcohol, “Even the smallest amount? People would argue about red wine.”

Men particularly viewed AAut as reliable and persuasive. The comprehensiveness of the messages and the content resonated, “Gives you the whole picture,” particularly for alcohol and weight gain, as some men did not know the connection, “Can make you fat? Strong message.” There was a general caution that “Recognize that people will always drink, but give a reason to cut down.”

### Substitution Tone

#### Helpful and Practical if Equivalent Substitutions

The substitution tone was generally seen as helpful and practical, particularly when there were specific examples given. There was a preference for “easy” and “simple.” And substitutions needed to be equivalent in terms of time, effort required, and cost, “Alternatives are okay but take more effort.” Fruit and vegetables FVSub were well-received and accepted, “helpful, give specific ideas, seems achievable.”

Some participants were just grateful to be reminded of alternative options in the JFSub message, “Tasty alternative gives us hope that we can still enjoy food.” Others said the substitutions themselves were not acceptable, plausible or appropriate, “Offering ideas makes me consider them. But apples for cake? Need viable options.” And the notion that healthier food could be tasty was not acceptable for some, “Not true! In many cases the healthy option is far less tasty.”

The effort required for substitution needed to be equivalent to resonate, “You can have an apple, cake or burger, but salads are too much effort for a snack.”

#### Alcohol Substitution Realistic, but Say Limit Not Stop

The alcohol substitution tone message ASub was appreciated as it was viewed as a realistic reminder to “limit intake” or strive for “moderation” rather than “stopping” drinking. Although most reacted favorably to the substitution tone, one woman said “while this statement is encouraging it is quite negative, makes me feel defensive.”

Men were likely to perceive the tone as appropriate but the content unreasonable, particularly the suitability of replacing alcohol with nonalcoholic beverage, “Do not ever offer me a mineral water instead of alcohol!” and there was a suggestion to consider the reasons for drinking, “A better vibe it still doesn't address the reasons are drinking.”

### Empathetic Tone

#### Empathy Resonates If It Acknowledged Previous Attempts

The empathetic tone was liked because it was achievable, true, about feelings, and not too forceful. All groups indicated that they liked being acknowledged, encouraged, and supported for their efforts. However, the relevance of the acknowledgement was important. For example, for fruit and vegetables FVE, “I like how it emphasizes feeling good, not just an unseen long-term health goals.”

JFE was seen as provoking and effective, “It challenges you not to follow the crowd” and “If I got this message, I would think twice about what I was eating.” The use of the capitalized word “NO!” in text gave a feeling of external support, “The capitalization of the word NO! is good as it motivates us to be strong.” and “Will make me think twice, feels like I have someone with me.” Others wanted a substitute suggestion rather than just a straight out NO!

Acknowledgment and recognition of previous attempt was appreciated, “Appeals to my conscience, I find myself in this position where I typically give in.”

#### Saying “Stop” to Junk Food Provoked a Defensive Reaction

Some reacted negatively to the JFE because they were already trying to cut down and thought it meant “never” to eat junk food and some defended the right to eat junk food, while some saw it as patronizing, “People can’t be strong all the time, I don’t like them saying every now and again.” and defended the right to have junk food, “There’s no harm in having junk food occasionally” or “Don’t tell me what to do.”

#### Thought Provoking but Not a Motivator for Alcohol

There was a general feeling that the empathetic alcohol message AE also needed substitutions. The factual content made it thought provoking and a good reminder, but not necessarily a motivator, “Gets me thinking about what I drink but probably wouldn’t stop me.”

Although many responded to the reference to losing weight, the “empty calorie” reference was difficult to understand and weight gain was not what people thought about when drinking, “Weight gain would stop me drinking as much.” Males aged over 25 years said people think about exercise not calories, “I wouldn't decline drink due to calories.”

### Solutions Tone

#### Acceptability

The solutions message tone acceptability was dependent on the behavior change proposed. The fruit and vegetables FVSol message was considered unrealistic, impractical, effortful, and costly, particularly the shopping, “Daily is unrealistic, weekly is better.”

#### Realistic and Helpful for Junk Food

The junk food solution JFSol was considered realistic, salient, practical, helpful, and good advice. It was liked because they encouraged preplanning meals with laziness, business, and forgetfulness the reasons given for not being prepared. Participants identified with message scenario, “I do get caught out. This one speaks to me.” The distinction was made between solutions and authoritative tones, “More of a healthy tip than an order.”

#### Should Encourage “Slowing Down” Not Stop Drinking

Participants were divided on the appropriateness of the alcohol solution ASol message, some people liked the mineral water substitution, but others, mostly men, thought it was boring or inappropriate. “Slowing down” rather than stopping was preferred as an appropriate solution, “Good, encourage slowing down, not completely stopping.”

#### Focus on “Maintaining” Not “Gaining or Losing” Weight

The “deceptively fattening” terminology motivated some women to think twice about drinking alcohol or to choose mineral water, but not so for men. The subtle orientation to weight status in the ASol message was acknowledged, “I like to focus on maintaining a healthy weight rather than not gaining or losing weight.”

#### The Food or Alcohol Calorie Trade-Off

More information was requested regarding alcohol solutions (eg, how the caloric content of alcohol compares to other junk food). Some men considered ASol interesting, effective, and providing good options while others did not like it. There also appeared to be a trade-off between eating healthy and alcohol intake, “If you are already trying to eat healthy then you don't need to worry about this.”

### “Gen Y” Tone

#### Either Friendly and Informal or Belittling

The Gen Y tone was considered least persuasive for fruit and vegetables FVGY, but had reasonable support for junk food and alcohol messages (Table 2). Depending on the behavior targeted, the Gen Y tone was seen as casual, friendly, humorous, catchy, and motivational with good ideas that made people stop and think. Some cautioned that Gen Y messages were at risk of trying too hard to be funny, being too colloquial and patronizing, particularly the reference to the white coat in the FVGY, “Belittling, feel I’m five years old!” Although the least favored tone to motivate fruit and vegetable intake, FVGY was valued as the “Use of informal tone stops it from sounding preachy.”

#### Either Defensive or Appreciative for Junk Food

Participants said the motivators for eating junk food were different to those for eating fruit and vegetables and there was a feeling that the JFGY was taking away small pleasures, “I don’t always feel bad and I don’t enjoy having to watch everything I eat” and “It [this message] takes the fun out of life.”

Some reacted negatively to the junk food JGY as they thought it was telling them how they felt or what to do, “I didn’t like you telling me how I feel” or “I feel like I have been smacked like a child. I would prefer to be spoken to on an adult level.” The discussion led to expressions of the need for freedom of choice, “While this is true it doesn’t really hit home, junk food is a personal choice.”

Even though the JFGY message tone was thought to be strong, “Strong message telling me not to, straight to the point” many liked it as they related to and believed it, “We do feel better when we leave it and eat healthy foods” and “We all know the TRUTH here, you do feel worse after junk!” Others wanted more information as they thought the recommendation to eat less junk food only related to people who need to lose weight, “Why is it bad? Not specific enough.”

#### Junk Food and Alcohol Gen Y Tone Strong but Resonated for Some

The directive message content for alcohol AGY was liked by some, “Nice and blunt” and “Like the reminder a friend would give” and others found it a “little rude” and were put off. It was seen by some as patronizing, “Sounds too much like it’s trying to imitate teenagers.” There were gender differences to the message, “You booze you lose is great!” or “I like it, it uses a well-known statement and adds a twist” from women whereas, “Stupid and misses the point” from males.

### Wording Preferences Across All Messages

Throughout the focus group discussions wording preferences and recommendations for incorporation into text messages for young adults were recorded, Table 5 presents the summary. The messages were then revised for use in a text messaging intervention and are outlined in Table 6 [19].

**Table 5:** Language suggested and preferred for nutrition messages by young adult focus group participants.

Language preferences	Fruit and vegetable	Junk food	Alcohol
Generally liked phrases	You need lots of fruit and vegetables to stay healthy	High fat, sugar and salt	Swap alcohol for juice
	Looking after yourself	We’re all tempted	Drink less
	You’ll find it’s easy	Quick fix	Alcohol is deceptively fattening—drink less
	You’ll feel great!	You really should be strong and say NO!	Limit your intake
	Healthy snacks	You know you’ll feel better if you…	Most people like a drink now and again
	It’s not hard	A few days’ fresh food in the house	Alcohol is a killer
	Swap roast veggies for chips	You’re prepared	You booze you lose
	Have a fruit salad for breakfast	Salads for snacks	
Generally disliked phrases	If you’re not getting…you are just not….	So avoid it	Useless energy
	Awesome	Coz	Pointless calories
	Nasty fatty stuff	It’s all bad	Empty calories
	The guys in white coats	Leave it alone	Health experts
	Daily	Make sure	If you insist on drinking…
		Always	…Offers you a drink
		Swap an apple for a piece of cake	drop the drink
		Swap vegges for chips	...Swap alcohol for mineral water

## Discussion

### General Influences

Participants described their food choices as being influenced by the following factors: the competing demands of work and study commitments leading to a desire for quick and convenient meals; the high cost of healthy food; and their feelings or emotions.

In terms of message content, they liked being acknowledged for steps already taken to improve their diet and did not like being asked to make unreasonable changes. Although there was awareness of, and in some cases disregard for, existing health recommendations and messages, there was a strong interest in the health consequences of dietary choices, particularly for excess alcohol and junk food consumption. Overall there was a preference for short, informative, and direct nutrition messages.

### Substitution Techniques and Empathetic Tone Favored Overall

Overall the technique of substitution and tone of empathy were favored as the most likely execution styles to motivate nutrition behavior change. Females were more likely to rate substitution messages persuasive compared to males; however, they disliked message content that implied restriction or failed to acknowledge previous attempts to change behavior. Males were more likely than females to favor the authoritative messages although it was not because of their tone but due to the rational reasoning within the message content. Positive, supportive or directive message tones appeared to be preferred overall. This is consistent with previous research which highlights the importance of message framing and that positively framed messages were generally favored [18,21].

Message communication research suggests that message persuasion potential is related to the message source, context, and the particular desired outcome [8]. This research found that there were differences in the preference for message techniques depending on the sought after nutrition behavior.

**Table 6:** Revised message for mobile phone intervention based on the findings of focus group with young adults.

Nutrition behavior change area	Target audience	Message
Fruit and vegetables	All	When you’re hungry you want to grab the closest thing in the fridge, so make sure it’s something healthy. Just cook up a bulk meal with veggies and freeze it. Something healthy ready when you are.
	All	Fitting veggies into your day can seem tricky, but why not just add some frozen peas or corn to your meal! It’s easy.
	All	Veggies can taste great if you know how to cook them. Try a quick and easy…
	All	Buying loads of veggies is great, but they’re not much use at the back of your fridge. Check out some of our quick, easy recipes that’ll get them out of your fridge and onto your plate.
	All	It’s easy to reach for unhealthy snacks when you’re hungry, but fruit makes great, quick snacks too. Keep some handy for when those hunger pangs hit.
Junk food	All	When you’re hungry, you need food now! But grabbing the nearest bit of junk food won’t do you any favors. Take a few minutes to find a tasty wrap or salad sandwich and you’ll feel much better.
	All	There’s never time to make lunch when you’re running late, and eating out often means eating junk. But a chicken salad or a fresh deli sandwich can be quick and healthy.
	All	If you haven’t sorted out your lunch for the day, healthy food is harder to find. So try planning ahead and pre-making something simple the night before to grab on your way out the door.
	All	We’re all tempted by fast food, even though we know it’s bad for us. If you can’t say no completely, just drop the chips or choose the meal with salad.
	All	One minute you want junk food but the next you wish you hadn’t. Eat regular healthy snacks and keep the cravings at bay.
Alcohol	All	Everyone likes a few drinks with friends, but too much alcohol and you’ll stack on the weight. So try and slow down your drinking.
	Women only	You’ve worked hard to fit into that party dress. But a (glass of wine has the same amount of kilojoules as a …). So try space your alcohol with water and keep looking great.
	Women only	Everyone deserves a fun night out. Just remember that alcohol’s high in kilojoules. By slowing your drinking you’re keeping in shape.
	Men only	Lots of people drink on the weekend, but drinking too often can make you feel slow and sluggish. Cutting out beers during the week is an easy way to feel more energetic.
	Men only	Every big drinker’s done something they’re embarrassed about. They might not remember it, but Facebook will. So try to slow down the drinking and space your drinks with water. It could save your reputation.
	Men only	Want to catch up with friends after work? Swap the pub for some sport. You’ll burn off the kilojoules instead of drinking them.

### Favored Tones Depended on Desired Behavior

Substitution was favored as the most persuasive message technique to encourage increasing fruit and vegetable intake. Messages for fruit and vegetables, known to the target group are likely to result in intention to comply [22,23]. The Department of Health in Western Australia, considered a credible and reliable information source, conducted high profile population based fruit and vegetable social marketing campaigns since 2000 [23]. When discussing the fruit and vegetable messages, the prescriptive *Go for 2&5* campaign message was often mentioned, confirming the high awareness of the recommendation reported elsewhere [23,24]. All communication executions for the *Go for 2&5* campaign were friendly, humorous, and a quirky, eg, advertisements with animated characters conducting cooking demonstrations offering quick and easy recipe suggestions and ideas [23]. The focus group discussions and message ranking showed preference for the communication style and ToV.

For junk food, offering solutions and empathic tone were favored as the most likely message techniques to encourage reduced intake. There was no clear message technique favored to reduce alcohol intake, in fact, the messages to limit or reduce intake did not appear to resonate. However, the discussions revealed that there was an interest in more specific information and statistics regarding longer-term health consequences and that the Gen Y and authoritative tones resonated.

There were gender preferences for ToV and message content for alcohol and weight control components. This finding is consistent with previous work that found obesity-related health messages may be perceived as stigmatizing and instilling less motivation to take action [22].

### Alcohol and Junk Food Tones Offensive yet Informative

There was confusion about junk food and alcohol recommendations and a feeling that the current health authority advice was either too strict, aspirational or missed the point. Participants were less certain of the negative impact, short or long-term, of consuming junk food or alcohol, and were interested in knowing more. The need for more information regarding the consequences of dietary change was a similar finding to formative evaluation undertaken for the *Go for 2&5* campaign, which found that young adults wanted to understand why they should eat more fruit and vegetables [24]. There appears to be a similar need for information to assist young people to make informed choices regarding junk food and alcohol.

Messages recommending action may need to explain why the action is important [5]. Prescriptive and/or descriptive information regarding recommended consumption and the health consequences of excessive intake of alcohol or junk food may be of value for young people, particularly as there was confusion about recommendations. Previous research suggests that information on the sugar content and potential health impacts of various beverages should be delivered in modes that are acceptable to young people [25]. The focus group discussions suggest that communications should focus on raising awareness and increasing knowledge and acceptance of the health risks of excessive junk food or alcohol consumption to change attitudes, possibly prior to any specific behavior change communication [7].

Individuals responded differently to message framing and content. Our current findings suggest that the empathetic tone would be most helpful when challenging young people to eat less junk food or limit drinking. Negative or defensive reactions to messages advising to limit or not consume alcohol or junk food were similar to previous Australian research testing text messages with obese adolescents [16]. Identifying and avoiding inferior or potentially counterproductive messages and tones may be as important as identifying the messages that are more likely to be effective. For many the advice was to frame alcohol and junk food messages to “limit” rather than “stop” and to focus on “maintaining” weight rather than “losing it”. Participants wanted to know “why” to drink less alcohol, but at the same time did not like to be told to “stop” drinking alcohol or eating junk food all together with support to help resist the “temptation to eat junk food” or drink too much.

### Inform and Suggest Appropriate Action to Engage

The message source, context, and personal relevance as well as the specific steps to changing dietary behavior were shown to be important. Overall, our findings are consistent with the CDC suggestions that in order to quickly engage the reader, messages need to be clear, give important information first, be action-based and easy to understand [5]. Personally relevant information is more systematically processed than less salient information, and attentive processing is required for effective health communications [10,26]. People are more likely to change their diets following personalized and specific nutrition messages [27,28]. This is evidenced by initial success in a mobile phone application providing automatic personalized messages to improve the user’s nutrition and physical activity behavior [29]. It is likely that short factual nutrition text messages and quizzes would be well-received with this audience, as they were with teenagers in the United States [17].

### Message Tone and Content Should be Tested for Each Behavior

Preference for message tone does not necessarily reflect its potential impact on attitudes or behavior as some of the least liked messages created the most interest, often a pre-requisites for behavior change [30]. The authoritative tone was the least liked but may be used to convey serious communications. Research suggests that over-simplifying plain language can be perceived as condescending by some consumer groups [9]. Although the Gen Y tonal messages were perceived as unusual, quirky, and fun by some, they were not viewed favorably by others. Social distance can be created or reduced based on language [9]. A conversational, informal position can come across as inauthentic and false, which appeared to be the case with the Gen Y ToV for some.

### Message Tone and Content in Whole Diet Context

Dietary behavior changes are not made in isolation, eg, when trying to decrease junk food intake, it would be likely that nutritious snacks such as fruit might be suggested as an alternative. The young adults in this study wanted practical, realistic, convenient, and low cost suggestions to support their dietary change. This is consistent with previous Australian research which found that when attempting to influence health behavior, time scarcity was a stress associated with limited economic resources [31]. These current findings reinforce that there is not a “one size fits all” healthy eating message that would motivate change across the population. The appropriateness and preference for the message tone differed with the nutrition behavior being addressed, readiness to change, perceived barriers, gender specific differences and individuals within groups responded differently. There is unlikely to be one nutrition message suitable for the general population as these types of differences between individuals rarely disappear [32]. However, the warm and approachable empathetic tone of voice should be considered in communications challenging current nutrition behaviors [9].

### Recommended Approach to Dietary Message Testing

The nutrition text message development was based on the framework for health campaigns proposed by Matterson et al as we (1) convened nutrition experts to agree on evidence that needs to be communicated; (2) convened health communication specialists to establish strategy and conduct formative research of message process; (3) implemented the findings; and (4) corrected the messages based on the outcomes for use in an intervention trial [7]. Table 6 presents the corrected messages which are to be used as part of a randomized controlled trial to improve eating behaviors via a mobile phone text messaging intervention [19].

The personal relevance of nutrition text messages influences their acceptability [5]. An unexpected finding was the similarity in opinions expressed between the genders, with the exception of discussion relating to alcohol messages or gender specific weight loss suggestions, for example about “fitting into that party dress”. Further research is needed to explore and quantify gender specific responses to messages challenging alcohol and junk food intake and weight status.

The findings of this research were part of formative evaluation for an intervention specific to young adults residing in Perth, Western Australia. Therefore, caution would be needed when using these with other groups. A limitation of the current study is that the ToV message testing was only applied to 3 nutrition messages, 15 messages in total, as time did not permit further messages being assessed or discussed. The focus group methodology enabled in-depth discussion and responses to these 15 messages and the approach provided insights into audience reactions to the message tone, content, and external factors influencing behavior change. We recommend that further work is conducted to specific nutrition behaviors (eg, to reduce confectionary intake or take away food). The approach of using a combination of qualitative and quantitative research to test dietary messages before conducting an intervention was useful. We plan to further develop and test the messages in a text messaging intervention with a wider audience [20].

#### Conclusion

Text messaging communications deliver health messages direct to individuals with the challenge of delivering salient and persuasive nutrition message content in 2-3 sentences. This research provided insights into the appropriate message ToV and content for text messages to promote dietary change for young adults. The technique of substitution and tone of empathy were favored as the most likely execution styles to motivate nutrition behavior change. Message development research is important for effective interventions and public health practitioners need to pay close attention to how the message will be received by the recipient.

## Abbreviations

A: alcohol
Aut: authoritative
AAut: alcohol authoritative
AE: alcohol empathetic
AGY: alcohol Gen Y
ASol: alcohol food solution
ASub: alcohol food substitution
E: empathetic
EDNP: energy dense nutrient poor
FV: fruit and vegetables
FVAut: fruit and vegetable authoritative
FVE: fruit and vegetable empathetic
FVGY: fruit and vegetable Gen Y
FVSol: fruit and vegetable solution
FVSub: fruit and vegetable substitution
Gen Y: generation Y
GY: generation Y
JF: junk food
JFAut: junk food authoritative
JFE: junk food empathetic
JFGY: junk food Gen Y
JFSol: junk food solution
JFSub: junk food substitution
Sub: substitution
Sol: solution
ToV: tone of voice

## References

[1] World Health Organization (2011). Global status report on noncommunicable diseases.

[2] National Health and Medical Research Council (2013). Eat for Health. Australian Dietary Guidelines. Providing the scientific evidence for healthier Australian diets.

[3] Hawkes C, Jewell J, Allen K (2013). A food policy package for healthy diets and the prevention of obesity and diet-related non-communicable diseases: the NOURISHING framework. Obes Rev.

[4] World Health Organization (2013). Global action plan for the prevention and control of noncommunicable diseases 2013-2020.

[5] Centers for Disease Control and Prevention (2012). CDC’s Guide to Writing for Social Media.

[6] Mobile Commons (2014). Why the humble text message is healthcare's secret weapon.

[7] Mattson M, Basu A (2010). The message development tool: a case for effective operationalization of messaging in social marketing practice. Health Mark Q.

[8] Shen L, Bigsby E (2012). The effects of message features: content, structure and style. The SAGE handbook of persuasion developments in theory and practice.

[9] Delin J (2007). Brand Tone of Voice. jal.

[10] Iversen AC, Kraft P (2006). Does socio-economic status and health consciousness influence how women respond to health related messages in media?. Health Educ Res.

[11] Whittaker R, Maddison R, McRobbie H, Bullen C, Denny S, Dorey E, Ellis-Pegler M, van Rooyen J, Rodgers A (2008). A multimedia mobile phone-based youth smoking cessation intervention: findings from content development and piloting studies. J Med Internet Res.

[12] Newton KH, Wiltshire EJ, Elley CR (2009). Pedometers and text messaging to increase physical activity: randomized controlled trial of adolescents with type 1 diabetes. Diabetes Care.

[13] Patrick K, Raab F, Adams MA, Dillon L, Zabinski M, Rock CL, Griswold WG, Norman GJ (2009). A text message-based intervention for weight loss: randomized controlled trial. J Med Internet Res.

[14] Woolford SJ, Derry HA, Jepson CM, Clark SJ, Strecher VJ, Resnicow K, Barr Kathryn L C (2011). OMG do not say LOL: obese adolescents' perspectives on the content of text messages to enhance weight loss efforts. Obesity (Silver Spring).

[15] Buchholz SW, Wilbur J, Ingram D, Fogg L (2013). Physical activity text messaging interventions in adults: a systematic review. Worldviews Evid Based Nurs.

[16] Smith KL, Kerr DA, Fenner AA, Straker LM (2014). Adolescents just do not know what they want: a qualitative study to describe obese adolescents' experiences of text messaging to support behavior change maintenance post intervention. J Med Internet Res.

[17] Hingle M, Nichter M, Medeiros M, Grace S (2013). Texting for health: the use of participatory methods to develop healthy lifestyle messages for teens. J Nutr Educ Behav.

[18] Lewis S, Thomas SL, Hyde J, Castle D, Blood RW, Komesaroff PA (2010). “I don't eat a hamburger and large chips every day!” A qualitative study of the impact of public health messages about obesity on obese adults. BMC Public Health.

[19] Kerr DA, Pollard CM, Howat P, Delp EJ, Pickering M, Kerr KR, Dhaliwal SS, Pratt IS, Wright J, Boushey CJ (2012). Connecting Health and Technology (CHAT): protocol of a randomized controlled trial to improve nutrition behaviours using mobile devices and tailored text messaging in young adults. BMC Public Health.

[20] Owen WF (1984). Interpretive themes in relational communication. Quarterly Journal of Speech.

[21] Kessels LT, Ruiter RA, Brug J, Jansma BM (2011). The effects of tailored and threatening nutrition information on message attention. Evidence from an event-related potential study. Appetite.

[22] Puhl R, Peterson JL, Luedicke J (2013). Fighting obesity or obese persons? Public perceptions of obesity-related health messages. Int J Obes (Lond).

[23] Pollard CM, Miller MR, Daly AM, Crouchley KE, O'Donoghue KJ, Lang AJ, Binns CW (2008). Increasing fruit and vegetable consumption: success of the Western Australian Go for 2&5 campaign. Public Health Nutr.

[24] Carter OB, Pollard CM, Atkins JF, Marie MJ, Pratt IS (2011). 'We're not told why--we're just told': qualitative reflections about the Western Australian Go for 2&5® fruit and vegetable campaign. Public Health Nutr.

[25] Hattersley L, Irwin M, King L, Allman-Farinelli M (2009). Determinants and patterns of soft drink consumption in young adults: a qualitative analysis. Public Health Nutr.

[26] Ruiter RA, Kessels LT, Jansma BM, Brug J (2006). Increased attention for computer-tailored health communications: an event-related potential study. Health Psychol.

[27] Brug J, Steenhuis I, van Assema P, Glanz K, De Vries H (1999). Computer-tailored nutrition education: differences between two interventions. Oxford Journals, Health Education Research.

[28] Pollard CM, Daly AM, Binns CW (2009). Consumer perceptions of fruit and vegetables serving sizes. Public Health Nutr.

[29] Rabbi M, Pfammatter A, Zhang M, Spring B, Choudhury T (2015). Automated personalized feedback for physical activity and dietary behavior change with mobile phones: a randomized controlled trial on adults. JMIR Mhealth Uhealth.

[30] Donovan R, Henley N (2003). Social marketing: principles and practice.

[31] Strazdins L, Broom DH, Banwell C, McDonald T, Skeat H (2011). Time limits? Reflecting and responding to time barriers for healthy, active living in Australia. Health Promot Int.

[32] Kitzinger J (1994). The methodology of Focus Groups: the importance of interaction between research participants. Sociol Health & Illness.

